# Guidance on interim analysis methods in clinical trials

**DOI:** 10.1017/cts.2023.552

**Published:** 2023-05-15

**Authors:** Jody D. Ciolino, Alexander M. Kaizer, Lauren Balmert Bonner

**Affiliations:** 1 Department of Preventive Medicine (Biostatistics), Northwestern University Feinberg School of Medicine, Chicago, Illinois, USA; 2 Department of Biostatistics & Informatics, Colorado School of Public Health, Aurora, Colorado, USA

**Keywords:** Interim analysis, clinical trials, randomized controlled trial, guidance, efficacy, futility

## Abstract

Interim analyses in clinical trials can take on a multitude of forms. They are often used to guide Data and Safety Monitoring Board (DSMB) recommendations to study teams regarding recruitment targets for large, later-phase clinical trials. As collaborative biostatisticians working and teaching in multiple fields of research and across a broad array of trial phases, we note the large heterogeneity and confusion surrounding interim analyses in clinical trials. Thus, in this paper, we aim to provide a general overview and guidance on interim analyses for a nonstatistical audience. We explain each of the following types of interim analyses: efficacy, futility, safety, and sample size re-estimation, and we provide the reader with reasoning, examples, and implications for each. We emphasize that while the types of interim analyses employed may differ depending on the nature of the study, we would always recommend prespecification of the interim analytic plan to the extent possible with risk mitigation and trial integrity remaining a priority. Finally, we posit that interim analyses should be used as tools to help the DSMB make informed decisions in the context of the overarching study. They should generally not be deemed binding, and they should not be reviewed in isolation.

## Introduction

The term “interim analysis” in clinical trials has multiple meanings. In general, interim analyses help guide decisions on overall clinical trial modifications, specifically those pertaining to the study sample size or recruitment targets [1,2]. The goal of the interim analyses will drive the decision on the type of analyses to conduct. Since the context of any given trial will determine the research objectives, study design, sample size, study outcome(s), and final analyses, these elements will also guide the appropriate interim analyses. An interim analysis for early-phase studies (phase I or early phase II) would often be linked to safety outcomes or adaptive designs methods, and thus they typically have differing goals than for later-phase trials. In general, early-phase studies usually involve small sample sizes and short follow-up and are often exploratory in nature, making interim analyses for early-phase studies often impractical. Therefore, we focus our discussion on the interim analyses that might be best used in larger, later phase (late phase II or phase III-IV), and often confirmatory trials.

Researchers must keep study integrity and bias mitigation a priority when planning, implementing, or interpreting interim analyses. Prespecification and impartial review of interim analysis are thus key in ensuring rigor. The study sponsor or lead investigative team will oftentimes enlist an external committee or board that can be impartial (i.e., not directly involved in the study design/conduct/analysis) to help interpret interim analyses and assist in decision-making based on these interpretations [3,4]. We will refer to these external boards as Data and Safety Monitoring Boards (DSMBs) for the purposes of this manuscript, noting that they are also known as Data Monitoring Committees or Data and Safety Monitoring Committees. When tasked with reviewing interim analysis results, DSMBs are meant to use them as a guide to make recommendations on potential study design adaptations to the sponsor and study team. We emphasize the notion of guidelines here as the DSMB should use interim analysis results as one piece of information and one tool in decision-making within the context of the whole picture of the trial. The DSMB should not use these guidelines in isolation to make interim recommendations to study sponsors and investigators [3].

While interim monitoring of things like processes, screening rates, visit adherence, study intervention adherence, etc., may also help with interim decision-making and ensuring overall trial integrity, we view this data quality monitoring as a separate issue [5–7] – much less prone to resulting in added biases – and we will not focus on this aspect of study monitoring in this manuscript. Rather, in this paper, we focus on interim analyses involving primary and key secondary outcomes that have potential to result in overall study design adaptations.

As collaborative biostatisticians working and teaching in multiple fields of research and across a broad array of trial phases, we note the large heterogeneity and confusion surrounding interim analyses in clinical trials. Thus, in the present paper, we aim to provide a general overview and guidance on interim analyses for a nonstatistical audience. In the sections to follow, we will discuss each of the following types of interim analyses: efficacy, futility, safety, and sample size re-estimation. Within each section, we (1) describe the purpose in more detail, (2) give a high-level view of statistical reasoning behind them, (3) provide an example study, and (4) discuss implications for researchers to consider when contemplating their use. The discussion in this manuscript focuses on analyses set in a frequentist framework rather than a Bayesian framework.

## Interim Analysis for Efficacy

### Purpose

The most frequent connotation pertaining to the term “interim analysis” revolves around early looks at the data with potential to stop a trial early for efficacy. The process involves a statistical hypothesis test on primary and potentially key secondary outcome(s) at some interim point in the trial, and if there is a large enough signal early in the study suggesting efficacy of one intervention arm over another, then it may be ethically imperative and most efficient to stop the study early. Stopping early and reporting on findings will allow for the investigational product to progress faster in the development process to reach the target clinical population sooner [3].

### Reasoning

**Figure 1.**
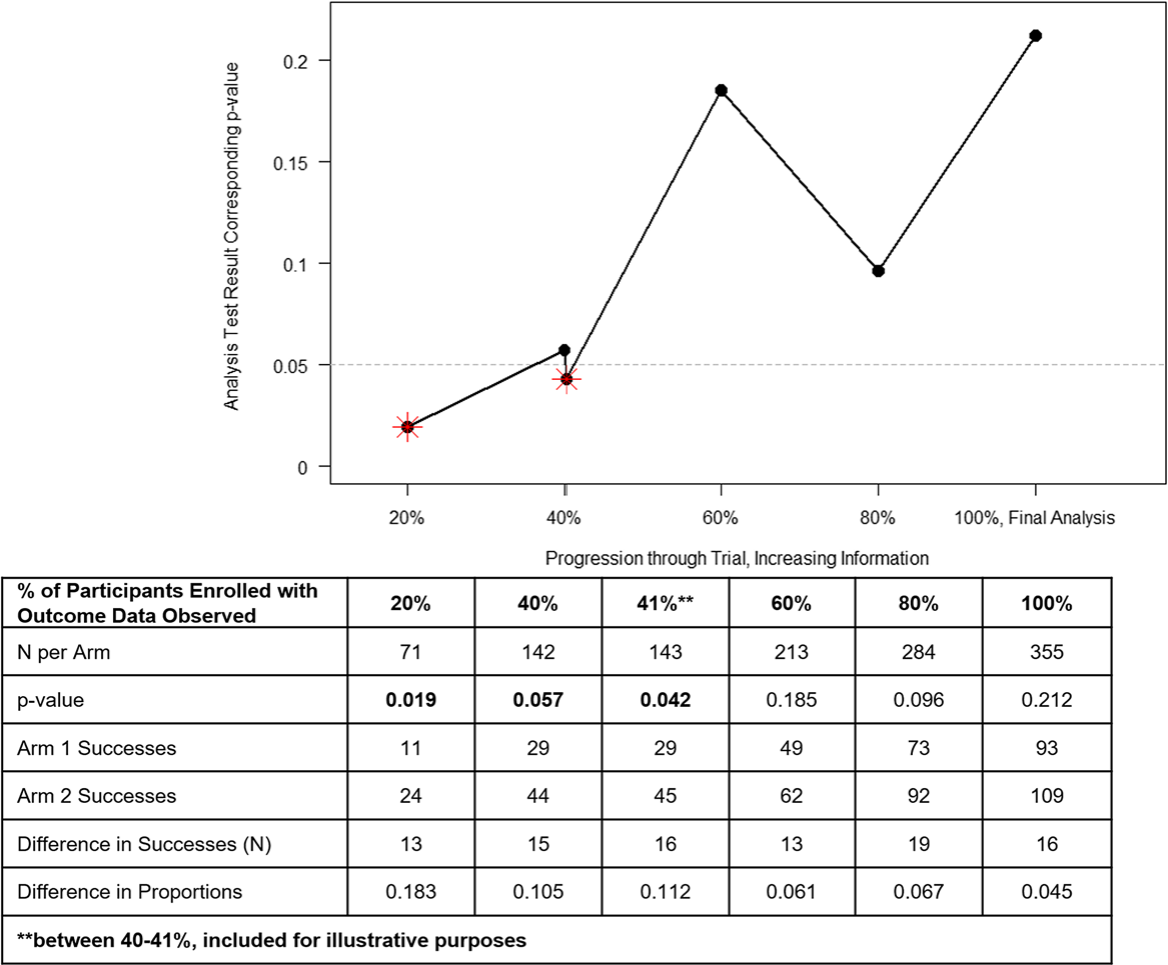
**Type I error illustration for a hypothetical null effect trial.** The data presented show a simulated, hypothetical two-arm clinical trial using a binary outcome, “success” of intervention. In the simulated example, there is no underlying difference in population proportions of successes across study arms, each set at a probability of success of 0.30. After randomly sampling observations from the two study arms, we conduct interim analyses for efficacy sequentially, at the shown information fractions. The starred data points indicate a statistical test result below the two-sided 0.05 level of significance. If we were to naively use the 0.05 threshold alone to make a decision to stop the trial based on an early efficacy signal, we would run the risk of incorrectly stopping at 20% of the way through the study, based on fairly unstable test statistics. The illustration at the transition between 142 (40%) and 143 (almost 41%; **included for illustrative purposes) participants per arm shows how easy it may be early on to move from an “insignificant” to “significant” finding with much less information than the overall sample would provide. With 143 participants, a difference on 16 participants (29 vs 45) experiencing a success across study arms corresponds to an 11% difference in proportions and a two-sided p-value of 0.042. Without protections on controlling type I error rate in a trial like this, we run the risk of incorrectly stopping the trial for early efficacy. At the end of the trial, the same 16-participant difference across arms results in an estimated 4.5% difference in proportions and an insignificant two-sided *p*-value of 0.212. We note this is just one of infinite possible example hypothetical trials.

The thresholds to use as sufficient evidence to stop a trial early remains subject to debate and requires careful consideration. Investigators cannot simply use a statistically significant (usually denoted as a two-sided *p*-value < 0.05) finding early on to guide this decision [1–3,8]. Recall the definition of type I error in an interventional study context: finding a statistically significant result when in fact there is no underlying intervention effect in reality. The more statistical tests we conduct, the more likely we are to find a significant result (i.e., make a type I error). Further, given the lack of stability of test statistics early in the trial, we must interpret statistical tests, conducted with less than the planned sample size, with increased scrutiny. Refer to Fig. 1 for a hypothetical example whereby we simulated a two-arm clinical trial with a null treatment effect and four interim analyses for efficacy. Since we simulated no intervention effect, we would hope the conclusion of the hypothetical trial would be that of no (significant) intervention effect. However, the plot in Fig. 1 illustrates the instability of test statistics, especially with small sample sizes. If we were to track its behavior after every participant, it may appear to bounce around, and as we get more information, it will stabilize and converge to its true value as the amount of information increases [9].

The methodology surrounding interim analysis evaluating for early efficacy signals thus centers on methods to control type I error and account for this sequential behavior of the test statistics throughout the course of the study. We usually set this type I error rate at 0.05, but this threshold is typically reserved for a single study analysis without a plan for interim analyses. The more analyses we do (including interim analyses), the more we need to adjust or correct for multiple hypothesis tests.

Interim analyses for early efficacy involve formal statistical hypothesis tests that mirror those prespecified in the primary analysis plan at the end of the study, and the methodology in this arena provides statistically justified guidelines to help researchers evaluate whether the resultant test statistic is “significant enough” such that it provides strong enough evidence to merit stopping for early efficacy. Among the most well-known methods for evaluation of these test statistics are group sequential methods [10–13] and alpha-spending functions [2,14,15] – sometimes these terms are used interchangeably, but there is a nuanced distinction.

**Figure 2.**
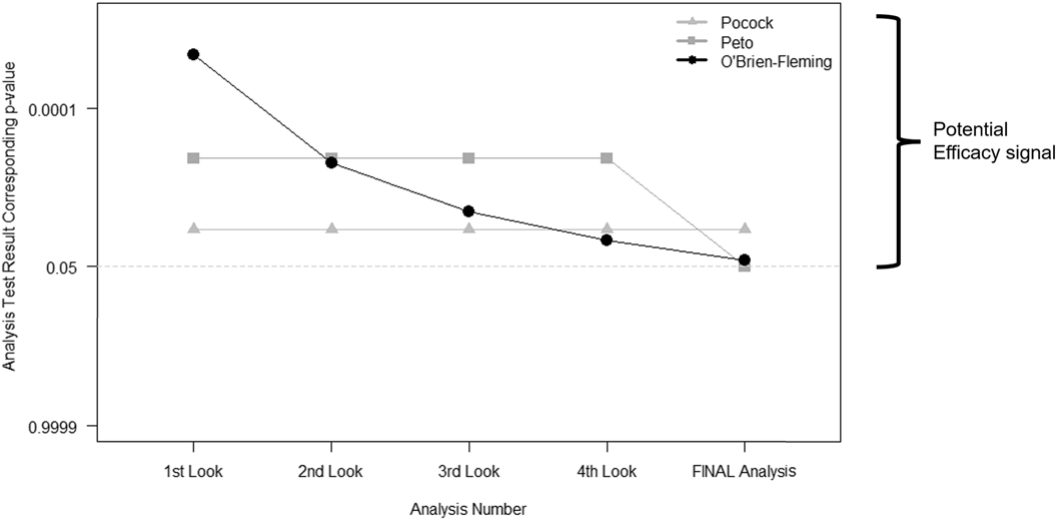
**Interim stopping bounds for group sequential methods with five evenly spaced analyses.** The plot provides a visual depiction of the three most well-known group sequential stopping bounds – Pocock, Peto, and O’Brien-Fleming – for a hypothetical clinical trial involving five total analyses: four interim analyses or looks and one final analysis, each equally spaced apart from one another. The faint dotted horizontal line provides a reference point for the typical two-sided *p* < 0.05 statistically significant result without any adjustment for multiple tests. Each method requires a more extreme result than the typical *p* < 0.05 for an investigative team and Data and Safety Monitoring Board to contemplate stopping for overwhelming efficacy. As illustrated, (a) the Pocock bounds have a constant approximate two-sided *p* < 0.016 threshold for all five analyses, (b) the Peto bounds have a stringent *p* < 0.001 threshold for the first four interim looks and *p* < 0.05 at the final look, and (c) the O’Brien-Fleming has very stringent thresholds early on, but the final analysis threshold is near the typical *p* < 0.05 at approximately *p* < 0.04.

Group sequential methods predate alpha-spending functions. They call for a prespecified number of interim looks at the study data. Then the thresholds used to determine whether the evidence warrants early stopping depend on the number of these interim looks. The most commonly used bounds fall into one of three categories: Pocock, Peto, and O’Brien-Fleming (Fig. 2) [10–13]. The difference between each lies in the weight that each time point in analyses may carry or how the type I error is “spent” throughout the trial.

The more contemporary methods of alpha-spending functions [2,14,15] build upon these ideas of group sequential methods, but they allow for more flexibility: (a) the time points at which the interim looks need not be exact nor equally spaced, but they are based on the information fraction – the amount of outcome data obtained divided by the total amount of outcome data that is planned for the trial; and (b) the investigators can assign weight to different information fractions according to a function. The link between the two sets of methods – group sequential and alpha-spending – lies in the ability to use an alpha-spending function that behaves like those illustrated above: a Peto-like, a Pocock-like, and an O’Brien-Fleming-like alpha-spending function.

### Example

The Thrombectomy for Stroke in the Public Health Care System of Brazil (NCT02216643) [16] is an example of a study that stopped early for efficacy. Investigators randomized (1:1) stroke patients with proximal intracranial occlusion across 12 sites in Brazil to either standard-of-care (SOC) or SOC plus mechanical thrombectomy (a one-time procedure), and the primary outcome was the modified Rankin scale, a measure of disability, at 90 days. The investigators planned interim analyses after 25, 50%, and 75% of trial participants had completed their 90-day follow-up, and they used the alpha-spending function approach [14,15], taking advantage of the flexibility in the exact trial fraction and the way alpha is spent throughout the trial. According to the prespecified analysis plan, the one-sided bounds for overwhelming efficacy at these time points were *p* < 0.0125 at 25%, *p* < 0.0161 at 50%, *p* < 0.0203 at 75%, and *p* < 0.0248 at final analyses. The trial stopped early for efficacy after 174 (25%) of the 690 planned participants completed 90-day follow-up, with an adjusted common odds ratio of 2.24 (95% confidence interval [CI]: 1.30–3.88; *p* = 0.004) in favor of thrombectomy. The investigators state that since the resulting statistic crossed the prespecified stopping boundary and at the recommendation of the DSMB and Steering Committee, they ended enrollment thereafter. At the time of halting the trial enrollment, a total of 221 (32% of planned total) had undergone randomization and were included in final analyses. The final reported adjusted common odds ratio was 2.28 (95% CI: 1.41–3.69; *p* = 0.001).

### Implications

Controlling type I error rate in clinical trial analyses using a correction (e.g., Bonferroni, Holm’s, etc.) [8] is undeniably important to bear in mind, as the more times we analyze our data (e.g., multiple primary outcomes), the more likely we are to find something significant. This notion becomes even more complex and more imperative in the context of interim analyses for efficacy as we run the risk of erroneously stopping the trial early. Without appropriate methodology, the overall type I error is inflated as we introduce interim analyses such that we have more opportunities to make an incorrect decision on the accumulating data. If we detect a large intervention effect early in the study such that analysis results suggest such a drastic change as stopping early, we would hope that if we were to continue the study through to the end, the probability of this early large effect attenuating toward a null effect would be negligible. In the example trial noted above, we see a trivial change in the estimated effect after reanalysis using all randomized participants, but the evidence in favor of thrombectomy strengthened. The group sequential methods and alpha-spending functions discussed here provide researchers with a tool to maintain some control over type I error, ensuring more stringent criteria for claiming an efficacious intervention earlier on in the study.

While the difference between the group sequential methods and alpha-spending functions may seem trivial, the alpha-spending approaches allow for both rigor and clear specification of these interim analyses while providing flexibility for the exact timing of these analyses [2,14,15]. For example, a statistical analysis plan may prespecify an interim analysis for efficacy using the O’Brien-Fleming like alpha-spending function at 50% of the way through the trial but allow for flexibility such that the information fraction need not be exactly 50% (e.g., it may be 53% if the timing of analyses and logistical constraints make this level of precision operationally difficult).

The O’Brien-Fleming bounds [11] are perhaps most intuitive as they “spend” very little type I error early on, ensuring very strict criteria for claiming early efficacy when we have less information, but then they become less stringent as more information is obtained in the course of the study – ultimately ending with a critical value that is very close to that which would be used without interim looks (i.e., close to the typical 0.05 type I error at the end; refer to Fig. 2). Finally, researchers must bear in mind that just as the final analysis plan should be prespecified in any trial, any interim analyses involving hypothesis tests and potential type I error “spending” should also be prespecified.

In the example trial exploring thrombectomy in stroke patients [16], the investigators were able to address their research question regarding their intervention with fewer participants than planned, equating to more efficient use of participant time and study resources. This is the heart of the reasoning behind interim analyses for early efficacy, but the intervention being studied should be far enough along in the process of development to justify a potential to study in fewer than planned representative patients, and we must keep in mind the lack of stability of test statistics with smaller numbers. For these reasons, we recommend reserving efficacy interim analyses for later phase, confirmatory studies that are prone to have large sample sizes and lengthy follow-up.

## Interim Analysis for Futility

### Purpose

An interim analysis incorporating a futility assessment is designed to assess whether a trial is likely to meet its objectives if continued to completion. In a traditional randomized clinical trial designed to detect a clinically meaningful treatment effect, futility suggests that observing a statistically significant result at the end of the study is unlikely. Stopping a futile trial early can increase efficiencies from cost, resource, and participant burden perspectives [2,3,8,17]. Given this reasoning, futility analyses are most appropriate in mid-late-phase studies enrolling larger sample sizes.

### Reasoning

A variety of statistical methods exist for estimating the futility of a trial at an interim time point [2,17]. A specified futility boundary ideally allows for a high probability of stopping early when there is not a true treatment effect and a low probability of stopping early when there is a true treatment effect. Various methods have been proposed for defining optimal boundaries, including group sequential methods (similar to those for efficacy bounds) or error-spending functions which allow greater flexibility in the timing of interim analyses.

Alternative approaches are based on estimating the probability of “success” of the trial under various frameworks. For example, conditional power is an estimate of the probability of seeing a significant effect at the end of a trial based on the current trend in the data and making specific assumptions about the trend for the remaining participants not yet enrolled. These assumptions generally include (a) the originally hypothesized effect, (b) the observed effect at the interim time point, and (c) the null effect. Predictive power is a similar concept that utilizes a Bayesian framework, updating prior assumptions of the treatment effect with observed data and averaging conditional power over this distribution. In both approaches, an *a priori* threshold is specified, such that the trial is deemed futile if conditional power or predictive power are low, typically less than 0.1–0.2 [18,19].

### Example

The Stroke Hyperglycemia Insulin Network Effort (SHINE) randomized clinical trial was designed to evaluate the efficacy of intensive glucose control during acute ischemic stroke [20]. Initial sample size calculations determined that 1400 patients were needed to provide at least 80% power to detect a clinically meaningful absolute difference in proportion of patients with a 90-day favorable outcome. The trial design incorporated four interim analyses to assess efficacy and futility after approximately 500, 700, 900, and 1100 patients had completed the 90-day follow-up. Efficacy and futility boundaries were based on an error-spending function method, controlling the overall probabilities of false negatives (type II error) and false positives (type I error). The statistical analysis plan indicated the test statistic and corresponding two-sided p-value thresholds for each interim analysis, such that stopping the trial early for futility would be considered if the p-value at the interim analysis crossed the specified threshold (*p*-value ≥ 0.949, 0.896, 0.652, and 0.293, respectively). The futility decision rules were deemed nonbinding, such that the decision to stop for futility could be overruled based on other pertinent information. Following the fourth interim analysis after enrollment of 1151 participants, the trial was stopped for futility. The primary manuscript concluded that there was no significant difference in the proportion with 90-day favorable outcomes in intensive compared to standard glucose control (20.5% in intensive arm vs 21.6% in standard arm).

### Implications

The benefits and limitations of interim futility analyses have been well documented [21–23]. Incorporating a futility assessment can increase the efficiency of the trial, allowing trials that are unlikely to meet their objectives to stop early ultimately reducing costs, preserving resources, and limiting patient burden. Particularly in large clinical trials or vulnerable patient populations, an interim futility assessment may be essential to prevent patients from being unnecessarily randomized to ineffective treatments. Despite common misconceptions, an interim analysis incorporating a futility assessment alone does not inflate the type I error. Futility can be assessed while preserving the overall probability of a false-positive result at the final analysis. It can, however, have implications on type II error, reducing the overall power of the study by stopping early. Stopping a trial early for futility can also introduce challenges [22] in the interpretation of results. A smaller than planned sample size may result in less precision around the treatment effect (i.e., wider CIs) and may introduce bias toward the null (i.e., a smaller observed treatment effect) [24]. Early termination also increases the risk for potential imbalance in baseline covariates and decreases the power for any secondary outcomes of interest. While a null effect may be observed for the primary outcome of interest, stopping early can prevent the ability to detect important differences in secondary end points. It may also be possible to observe low conditional power (or small test statistic/large p-value) at an interim analysis that is driven by opposite treatment effects occurring in different subgroups [23]. As such, special attention should be given in scenarios where there is a plausible heterogeneity of treatment effect. A similar argument can be made in the presence of largely influential baseline variables that may exhibit large imbalances at the time of interim analyses [25].

The decision to incorporate an interim futility assessment should be made with careful consideration, evaluating the effects of futility thresholds on overall trial operating characteristics. When a futility analysis is deemed appropriate, it is recommended to strike a balance between prespecified rules and the flexibility to incorporate new information as the trial progresses. In general, futility analyses can be deemed binding or nonbinding. Binding futility rules are less common and not recommended as they require that a trial be stopped if the futility boundary is crossed, regardless of any other information. Nonbinding futility thresholds, as incorporated in the SHINE trial, allow for incorporation of other factors (such as secondary end points) or external information (such as information from related trials), and these are recommended over the binding rules [26]. Investigators should lean on DSMBs to provide independent and unbiased recommendations [3,4]. Regardless, transparency in reporting planned versus unplanned interim analyses and any decision rules is imperative.

## Interim Analysis for Safety

### Purpose

An interim analysis incorporating a safety assessment is designed to assess whether there is evidence for increased risk of adverse events in intervention study arms relative to the SOC arm or historic data. All studies, regardless of phase (e.g., Phase I, II, III, or IV), should incorporate safety monitoring. If safety concerns are identified, a study could either temporarily pause enrollment while investigating the potential causal nature between an intervention and adverse events or terminate the trial prior to full enrollment [1].

### Reasoning

Safety monitoring is done to protect the interests of trial participants so that they are not exposed to unnecessary risk in the presence of limited benefit. While serious and potentially unexpected adverse events may require immediate reporting to the DSMB and/or regulatory authorities and may result in trial modifications, this discussion focuses on the process of benefit-to-risk assessment. To determine the *benefit-to-risk* assessment, a safety interim analysis must be paired with an interim evaluation of the efficacy of the study’s primary outcome. Without efficacy information, it can be challenging, if not impossible, to contextualize the safety concerns.

The population enrolled in a study may also warrant additional safety considerations. For instance, the US Food and Drug Administration has designated special populations that warrant special attention, including pregnant women, children, prisoners, those with impaired cognition, and the elderly [27]. The DSMB, or other parties monitoring the study, should be concerned about the benefit-to-risk assessment in the context of these special populations when appropriate. In single-arm trials or studies without a SOC comparator, such as those for rare diseases or in oncology settings, historic rates of adverse events may be used as the reference to determine the benefit-to-risk ratio [28]. However, the potential for time biases, where the adverse events or condition studied itself are changing over time, need to be carefully contemplated when selecting and interpreting the historic reference data [29]. The comparison to a concurrently enrolled SOC arm may also be more useful to contextualize safety concerns because it is possible that increased rates of adverse events would be observed in both arms, indicating the study intervention may not be the primary cause [30].

### Example

The EARLY trial (NCT02569398) [31] was a three-arm, randomized, double-blind, placebo-controlled multinational phase 2b/3 trial exploring the short-term effects of atabecestat at two different doses compared to placebo in preclinical Alzheimer’s disease (AD). The primary efficacy outcome was the change from baseline in the Preclinical Alzheimer Cognitive Composite score. The planned enrollment was 1650 participants from 143 sites. While interim monitoring for futility was specified in the study protocol, no formal efficacy monitoring was planned. However, the trial was terminated early for safety after 557 participants were randomized due to hepatic safety concerns relating to serious elevations of liver enzymes. Based on the accumulated evidence, it was decided at the interim analysis that the benefit-to-risk assessment offered by the drug did not support its continued study. While study drug dosing was immediately halted, participants were followed off-treatment for 6 additional months to evaluate for any persistent safety concerns. The primary manuscript concluded that atabecestat would not be developed further given the safety concerns and confirmed dose-related worsening of cognition within 3 months of treatment initiation.

### Implications

The monitoring of safety outcomes and adverse events is paramount to maintaining the integrity of any study. The importance and understanding of safety monitoring has been discussed and developed from a wide variety of perspectives, including patients, investigators, DSMBs, and statisticians [3,32–36]. The choice to stop a trial early for safety concerns should be made in the context of the benefit-to-risk ratio, where the presence of some adverse events may become less tolerable and acceptable as the potential efficacy decreases. In trials where the primary outcome is a safety outcome, traditional group sequential methods may be used to monitor for interim differences.

While safety monitoring may raise concerns about multiple looks at the data before the final analysis, the potential benefit of the treatment is needed to determine if any increased risk of adverse events may be acceptable as a trade-off for improved efficacy. Further, if only safety monitoring is desired, interim efficacy boundaries may be set to fixed, small α-levels (e.g., <0.0001) or a conservative α-spending approach (e.g., O’Brien-Fleming as mentioned in efficacy monitoring) can be selected so that the impact on the final analysis is negligible [3].

The context of a given research study is also important, since some adverse events may not be unexpected in certain populations. For example, a cardiovascular secondary prevention trial to prevent subsequent myocardial infarction may have the expectation of some myocardial infarction events, whereas it may be extremely concerning to observe the same events in a behavioral intervention in a generally healthy population. In cases where there is suspected harm to study participants, the trial should carefully determine if any potential benefits are worth the increased and potentially serious risks to participants and future patients. If both hypothetical examples of a cardiovascular or behavioral study exhibit efficacy signals early on in conjunction with these safety signals, the benefit-to-risk ratio would be different for each trial because of the context. For that reason, there is no “one-size-fits-all” benefit-to-risk ratio guidance.

In practice, safety monitoring should be coupled with summaries of the efficacy of the primary outcome and with reference to a SOC arm or historic data to fully understand the benefit-to-risk ratio. Ultimately, depending on the safety concerns identified, a trial may temporarily pause enrollment to better assess causality or may terminate early due to a poor benefit-to-risk ratio where any benefit provided by the intervention is not identified as outweighing increased safety risks. These decisions are often best made through the independent DSMB and their review of the full picture to the extent possible, to providing informed and unbiased recommendations

## Interim Analysis for Sample Size Re-Estimation

### Purpose

A study with an interim analysis to re-estimate sample size is designed to modify the planned sample size based on the accumulating data within the trial to account for any uncertainty when conducting power calculations during the initial planning of the study. These approaches facilitate a revised sample size calculation – often through an internal pilot study [37] – using information for the ongoing assessment of event rates, the estimation of nuisance parameters (e.g., the variance of a continuous outcome), or the effect size expected [38]. Re-estimating a sample size at an interim stage can increase the likelihood of a successful trial but may result in a substantial increase in the needed sample size if the initial sample size assumptions were very different from what is observed.

### Reasoning

A range of statistical approaches have been developed to accommodate a variety of settings for uncertainty in clinical trials that may wish to adaptively re-estimate their sample size. The intention of these methods is to improve confidence that the present trial is adequately powered as more information is obtained. The statistical methods used for sample size re-estimation fall into two categories: blinded or unblinded [39]. The blinded or unblinded distinction is with regard to the study arm allocation of currently randomized participants. *Blinded* sample size re-estimation methods are primarily used to revise the estimation of nuisance parameters in a trial, such as the variance of a continuous outcome. These may use either a pooled estimate of the variance by combining all arms or use statistical approaches to incorporate variance estimates from multiple study arms [40–42].

In contrast, *unblinded* sample size re-estimation approaches are based on comparative interim results. These designs are ideal when there is uncertainty in both the estimates of the true effect size and the nuisance parameters to be measured. This adaptation allows the trial to capture an effect that may still be clinically meaningful but differs from the initial assumptions. Numerous statistical approaches have been proposed for sample size re-estimation with the goal of maintaining the desired type I error rate after having a comparative interim analysis [43–46].

### Example

The Tenecteplase versus Alteplase before Endovascular Therapy for Ischemic Stroke (EXTEND-IA TNK) trial [47] was a multicenter, randomized, open-label, non-inferiority, blinded-outcome trial that enrolled patients with ischemic stroke within 4.5 hours after onset and were eligible to undergo intravenous thrombolysis and endovascular thrombectomy. Participants were randomized 1:1 to either intravenous tenecteplase or alteplase. The primary outcome was the proportion of participants with restoration of blood flow to > 50% of the affected arterial territory or absence of retrievable thrombus at initial angiogram. The initial power calculation suggested a minimum sample size of 120 participants would have 80% power, but there was substantial uncertainty over the participant disposition within the trial and prevalence of the outcome [48]. A blinded adaptive sample size re-estimation approach was implemented after enrollment of 100 participants, and the re-estimated sample size was 202 participants to establish non-inferiority. The trial then continued to enroll a total of 202 participants, with 101 assigned to each study arm to ultimately determine that tenecteplase (22% event rate) was non-inferior to alteplase (10% event rate).

### Implications

Underpowered studies happen often and result in participant and resource waste [49], much of which may be mitigated with more certainty around assumptions in the initial power calculation. The ability of sample size re-estimation approaches to better ensure an adequately powered study increases efficiency for clinical trials. The higher likelihood of detecting a clinically meaningful effect, if it exists, may better utilize resources and can ensure that the time and contribution of trial participants are not wasted. Blinded re-estimation approaches generally have a limited effect on the type I error rate but may require additional steps to maintain trial integrity for methods which involve treatment assignment information [38]. In contrast, uncontrolled unblinded re-estimation may have a substantial impact on the overall type I error rate, where the desired alpha level may be doubled at the end of the trial without using appropriate preplanned methods [50]. However, numerous statistical methods have been proposed to maintain the overall type I error rate across a range of trial designs and outcome types [43–46].

There are practical considerations for choosing a sample size re-estimation approach. In our experience, increases in sample size are more common than decreases in sample size as a result of interim sample size re-estimation. It is possible that the re-estimated sample size is so large that it is infeasible due to resource or time constraints. If a sample size re-estimation is planned, the investigators should take care to determine a maximum possible sample size given available resources ahead of time and with consideration of a minimal effect size. In some cases, an unblinded re-estimation may identify a smaller effect size that may not be agreed upon as clinically beneficial by the broader research community and call into question the equipoise of a continued trial that could stop for futility. It is also possible that a trial could stop early for efficacy if there is an unforeseen large interim signal based on efficacy analyses, in line with more traditional group sequential methods to monitor for efficacy as discussed previously [51]. Care should be taken in how the results of a re-estimated sample size are reported for ongoing studies, since it may be possible to back-calculate the effect size if one knows the conditional power or other assumptions [38]. In all of these cases, prespecified rules for how to modify the sample size and what do to in different scenarios should be incorporated into the trial documentation.

## Discussion

Interim analyses using current data from an ongoing randomized trial can guide decisions on early study termination or modifications to the originally proposed sample size. Analyses conducted with the potential to alter the trial conduct include interim analyses for efficacy, futility, safety, and sample size re-estimation. Table 1 provides a summary of the interim analyses discussed.

**Table 1.** Summary of interim analysis types

Type of interim analysis	Explanation	Justification for use
**Efficacy**	Early termination of a trial that is showing promising results Control of type I error through group sequential methods or alpha-spending functions	Usually for longer, larger studies and later phases of research Ethical imperative for a promising treatment to reach the entire target clinical population
**Futility**	Early termination of a trial that is not likely to achieve the intended objective (e.g., little chance of finding a “significant” treatment effect at the end of the study) Employed through group sequential methods, error-spending functions, conditional power, or predictive power	Reduces costs, resources, and patient burden for a trial with a low probability of “success” Usually for mid-late-phase studies Helpful in the context of recruitment and retention challenges
**Safety**	Early termination (or pausing) of a trial for safety concerns Should be coupled with efficacy analyses to evaluate the benefit-to-risk ratio	Incorporated across all phases of research Particularly important for vulnerable populations and high-risk interventions with more “serious” outcomes (e.g., death)
**Sample size re-estimation**	Reassessment of the sample size required to ensure adequate power using updated information from interim trial data Can be blinded or unblinded May not necessarily spend alpha	Allows for interim look at assumptions (standard deviations, event rates, correlations, etc.) May be particularly useful for mid-late-phase studies

While the types of interim analyses employed may differ depending on the nature of the study, prespecifying the interim analytic plan to the extent possible is always recommended to mitigate risk of biases and maintain overall integrity of the study. Modifications to the analysis plan or protocol that were not planned will be met with larger scrutiny than modifications that were expected. Detailed documentation should describe anticipated timing of interim analyses, proposed statistical methodology, and any prespecified rules or thresholds to guide decisions. The timing of analyses can be flexible and is often specified when some proportion of participants is enrolled and meet a particular study milestone (e.g., 50% of participants completed 6-week follow-up). Care should be taken to strike a balance between having maximal information (later interim analysis) versus ensuring adequate time to make any modifications and reducing potential risk to participants as much as possible. Procedures for ensuring blinding of interim data and results, as appropriate, should also be documented. Interim results should be kept strictly with the DSMB and the unblinded study statistician. Only high-level recommendations from the DSMB and/or modifications to the trial should be communicated to the study team or external entities.

Before proposing an interim analysis plan, investigators should carefully think about potential logistical implications. For example, if an interim sample size re-estimation is proposed, are there adequate resources to support an increase in sample size if indicated? Simulation studies may be used in advance to explore possible scenarios and weigh pros and cons of any analyses of primary and key secondary end points.

Evaluation of interim analysis results should not be interpreted in isolation, but rather in the context of other internal study factors and external contemporaneous issues, including information that becomes available on outcomes, therapies, or within the relevant disease area in other studies. Any interim analysis results and statistical tools are intended to serve as guidelines. Independent and unbiased expertise from the members of the DSMB should be leveraged to inform decisions. Transparency in disseminating trial results when interim analyses were conducted is also critical. Final results should be interpreted in the context of any preplanned or ad hoc analyses during the trial.
